# Chinese Herbal Formula (CHF03) Attenuates Non-Alcoholic Fatty Liver Disease (NAFLD) Through Inhibiting Lipogenesis and Anti-Oxidation Mechanisms

**DOI:** 10.3389/fphar.2019.01190

**Published:** 2019-10-15

**Authors:** Yizhe Cui, Renxu Chang, Tao Zhang, Xiaocui Zhou, Qiuju Wang, Haiyun Gao, Lintong Hou, Juan J. Loor, Chuang Xu

**Affiliations:** ^1^College of Animal Science and Veterinary Medicine, Heilongjiang Bayi Agricultural University, Daqing, China; ^2^Open Project Program of Beijing Key Laboratory of Traditional Chinese Veterinary Medicine, Beijing University of Agriculture, Beijing, China; ^3^Laboratory of Zoonosis, China Animal Health and Epidemiology Center, Qingdao, China; ^4^Department of Animal Sciences and Division of Nutritional Sciences, University of Illinois, Urbana, IL, United States

**Keywords:** nonalcoholic fatty liver disease, herbal formula, hepatocytes, NF-κB, high-fat diet

## Abstract

Nonalcoholic fatty liver disease (NAFLD) is a hepatic ailment with a rapidly increasing incidence in the human population due largely to dietary hyper nutrition and subsequent obesity. Discovering effective natural compounds and herbs against NAFLD can provide alternative and complementary medical treatments to current chemical pharmaceuticals. In this study, ICR male mice were fed a high-fat diet (HFD) *in vivo* and the AML12 cells were treated with palmitic acid (PA) *in vitro*. We explore the protective effect and potential mechanism of Chinese Herbal Formula (CHF03) against NAFLD by HE staining, transmission Electron Microscopy assay, Western blotting, and gene expression. *In vivo*, oxidative stress markers (GSH, GSH-px, MDA, SOD, and CAT) confirmed that CHF03 alleviated oxidative stress and abundance of NF-κB proteins indicating a reduction in inflammation and oxidative stress. The lower protein abundance of ACACA and FASN indicated a preventive effect on lipogenesis. Histological and ultrastructural observations revealed that CHF03 inhibited NAFLD. Expression of *Srebf1*, *Fasn*, and *Acaca*, which are associated with lipogenesis, were downregulated. *In vitro*, genes and proteins are expressed in a dose-dependent manner, consistent with those in the liver. CHF03 inhibited lipid accumulation and expression of NF-κB, nuclear transfer, and transcriptional activity in AML12 cells. The CHF03 might have a beneficial role in the prevention of hepatic steatosis by altering the expression of lipogenic genes and attenuating oxidative stress.

## Introduction

One of the most serious health problems is liver disease, which affects more than 10% of the world’s population (Hong et al., 2015). The most common type of liver disease in industrialized countries is nonalcoholic fatty liver disease (NAFLD). Survey data indicate that NAFLD is the leading cause of chronic liver disease in the United States alone (more than 75%) (Younossi et al., 2011). Moreover, unlike other major causes of mortality, liver disease rates are increasing rather than declining (Than and Newsome, 2015).

According to the period and severity of liver injury, the types of liver injury can be divided into steatosis, steatohepatitis (Khalatbari-Soltani et al., 2019), fibrosis, cirrhosis, and cancer (Byrne and Targher, 2015). Normally, NAFLD is caused by nutrient factors and lipid accumulation in biological tissues (Donnelly et al., 2005). Recently, studies have shown that mitochondrial dysfunction is associated with the pathogenesis of NAFLD. Moreover, nuclear receptors and transcription factors involved in reactive oxygen species (ROS) are also regulation of liver lipid metabolism (Gusdon et al., 2014). In addition, decreased fat production and lipid clearance in the liver can also lead to liver steatosis and lipid-related liver damage (Musso et al., 2009).

At present, the therapeutic effect of synthetic drugs on NAFLD is not ideal, and most of them have adverse side effects (Gupta and Lewis, 2008). Therefore, it is urgent to develop new approaches to prevent and treat NAFLD. In recent years, many herbs and phytochemicals (Li et al., 2014) have been used as complementary and alternative therapies for liver diseases, including NAFLD (Wong et al., 2013). However, the efficacy of these herbs needs to be scientifically verified. In traditional Chinese medicine theory, the main symptoms of NAFLD include anorexia, chronic fatigue, abdominal pain, intestinal spasm, and nausea (Fan et al., 2017). We searched the literature of traditional medicine for herbs with anti-inflammatory, antioxidant, and lipid-lowering properties, and selected modified Sijunzi decoction, *Scutellaria barbata D. Don*, *Rehmannia glutinosa (Gaertn.) DC*, and *Glabrous greenbrier rhizome*. These have been frequently prescribed for treatment of the liver-related diseases and symptoms above.

*Scutellaria barbata D. Don* is known as Ban-Zhi Lian in Traditional Chinese medicines which has been used to inhibit inflammatory (Yang and Wei, 2018) and block tumor (Chen et al., 2017) growth. *Rehmannia glutinosa (Gaertn.) DC* is effective in treating patients with various inflammatory (Kim et al., 2017) and metabolic diseases such as high blood pressure and diabetes (Lv et al., 2016). *Cuscuta chinensis Lam* are commonly used as a medicinal material for treating aches and weakness of the loins and knees, tonifying the defects of the liver and the kidney, and treating diarrhea due to hypofunction of the kidney and the spleen (Liao et al., 2014). Sijunzi decoction, which is also known as the “Four Gentlemen” decoction, consists of four types of herbs: *Glabrous greenbrier rhizome*, *Panax ginseng C.A.Mey*, *Glycyrrhiza uralensis Fisch. ex DC*, and *Atractylodes macrocephala Koidz* (Wang et al., 2013). Recent reports have indicated that Sijunzi decoction inhibited the activation and expression of NF-κB p65 (Lu et al., 2018). The ratio of the different components in the decoction might affect the outcomes in different clinical indications. However, the mechanisms remain unclear.

In this study, we fed mice with high-fat diet and screened Chinese herbal prescriptions by HE staining. We formulated Chinese Herbal Formula (CHF03) a combination of 10 herbs, which is known to have an effect on liver-associated diseases in traditional medicine. We have provided, for the first time, some scientific evidences towards the efficacy of CHF03 in protecting against NAFLD. Furthermore, in the mechanistic study, CHF03 displayed the function of lowering lipid, antioxidant stress, and stabilizing mitochondria in the mice model of NAFLD. The present results revealed that CHF03 might be a potential NAFLD therapeutic agent.

## Materials and Methods

### Herb Materials and Preparation

The CHF03 formulation is shown in **Table S1**. Basis of the Chinese Pharmacopoeia (The Pharmacopoeia Commission of PRC, 2010), the herbal mix was purchased from Fu Rui Bang Chinese Medicine Co., Ltd. (Daqing, China). The CHF03 (total 144 g) was made into a water concoction containing 1 g/ml extract of herbal medicine (Cui et al., 2018b). The decoction was autoclaved at 100°C for 20 min and stored at 4°C.

### LC/MS Analysis

The samples were thawed at room temperature, and 100 µl of them was then transferred into Centrifuge Tubes (1.5 ml) by pipette. The decoction was extracted with 300 µl methanol and 10 µl internal standard (3 mg/ml, dl-o-chlorophenylalanine) was added. Samples were treated with 4 kHz ultrasound in ice bath for 30 min, rotated for 30 s, 12,000 rpm, centrifuged at 4°C for 15 min. The supernatant was transferred to a small bottle for LC-MS analysis (Cui et al., 2018a). Then 200 µl of supernatant was transferred to vial for LC-MS analysis. Analysis platform: LC-MS (Thermo, Ultimate 3000LC, Obitrap Elite) Column: Waters ACQUITY UPLC HSS T3column (2.1 mm × 100 mm, 1.8 µm) Chromatographic separation conditions: Column temperature: 40°C; Flow rate: 0.3 ml/min; Mobile phase A: water+0.1% formic acid; Mobile phase B: acetonitrile+0.1% formic acid; Injection volume: 4 µl; Automatic injector temperature: 4°C. The data was performed feature extraction and preprocessed with Compound Discoverer software (Thermo), and then normalized and edited into two-dimensional data matrix by excel 2010 software, including Retention time (RT), Compound Molecular Weight (compMW), Observations (samples), and peak intensity.

### Animal Treatment

All animal experiments were conducted in accordance with the “Guidelines for the Nursing and Use of Laboratory Animals” (NIH) and the rules and ethical standards formulated by the Ethics Committee of Heilongjiang Bayi Agricultural University. Six- to eight-week-old ICR male mice [Certification No: SYXK (HEI) 2014005] were purchased from the Animal Laboratory Center of Harbin Medical University (Daqing). Six mice were assigned to each of three groups. After a week of adaptation, the control group was fed a standard diet (12% kcal fat), the NAFLD group was fed a HFD (60% kcal fat) (68.5% standard diet, 15% lard, 1% cholesterol, 0.5% bile and 15% dextrin) according to literature (Cui et al., 2013), and the CHF03 group was fed HFD along with 10 g/kg CHF03 given orally (0.1 ml per 10 g body weight). After 8 weeks on treatments, mice in all groups were sacrificed *via* carbon dioxide asphyxiation. Subsequently, blood was collected *via* heart puncture, serum was centrifuged, and liver harvested for subsequent analyses. Food intake and body weight were measured daily and weekly, respectively.

### Biochemical Analyses

Each blood biochemical index was estimated using an automatic blood chemical analyzer (Hitcahi, Tokyo, Japan). All blood indexes were tested by Jilin University Animal Hospital.

### Oxidative Stress Analyses

Approximate amount of frozen liver homogenates were used for biochemical assays as described previously (Kennedy-Feitosa et al., 2016). Superoxide dismutase (SOD) activity was assayed by monitoring adrenaline (Sigma-Aldrich, USA) inhibition autooxidation. Catalase (CAT) activity was measured by a decrease of H_2_O_2_ (Sigma-Aldrich, USA) rate, and concentrations were monitored. As an index of oxidative damage induced by lipid peroxidation, we used thiobarbituric acid reactive substances (TBARS) (EMD Millipore, USA) method to analyze malondialdehyde (MDA) products during an acid-heating reaction. TBARS levels were expressed as MDA equivalents. Glutathione (GSH) and Glutathione peroxidase (GSH-Px) were determined using spectrophotometric assay kits. All produces were performed according to the manufacturer’s instruction.

### Liver Histological Examination

Fresh liver sections were fixed in 10% neutral formalin, embedded in paraffin, and stained with H&E (Dixon et al., 2004). Tissues were scored according to the criteria for evaluating changes in fat and inflammation. Pathologists examined sections to detect the presence of fat, necrosis, fibrosis, and inflammation (Kleiner et al., 2005).

*Oil Red O*. Samples were embedded in paraffin for Oil Red O staining (Reis et al., 2014). Liver tissue was fixed with 10% formalin for 3 days and processed by automatic tissue processing machine, then embedded in paraffin. The thin sheet (5 μm) was obtained and mounted on the glass slide. To detect accumulation of neutral lipids, sections were stained with Oil Red O for 15 min, repeated stained with Mayer vessels 5 times, and then covered with DPX. The slides were observed under an optical microscope. Microscopic photographs of the liver were taken.

### Ultrastructural Analyses

Liver samples were fixed in 2.5% glutaraldehyde in 0.1 mol/L cacodylate buffer, post-fixed in 1% osmium tetroxide, and embedded in Epon (Embed-812, USA). Ultra-thin sections (80 to 100 nm) were obtained from selected areas using an ultramicrotome (Leica UC7, Germany), contrasted with uranyl acetate and lead citrate, and examined with a transmission electron microscope (TEM) (Tecnai G2 20 ST, FEI, USA) (Felix et al., 2015).

### Cell Culture

The AML12 cells were kindly provided by Stem Cell Bank, Chinese Academy of Sciences and cultured according to established protocols. The medium consisted of Dulbecco modified Eagle’s medium and Ham’s F12 1:1 medium supplemented by 10% fetal bovine serum, penicillin-streptomycin (100 U/ml), 37°C, 95% air, and 5% carbon dioxide (Park et al., 2015).

### Preparation of Palmitic Acid Solution

Sodium hydroxide particles are 0.16 g soluble in distilled water at a constant volume of 10 ml, and are configured into 400 mM sodium hydroxide. The palmitic acid (PA) powder 0.1025 g and 400 mM sodium hydroxide 1ml were placed in a 50 ml centrifugal tube, heated in a 90°C water bath, and shaken gently until dissolved into a clear liquid to form a 400 mM PA solution. One gram of fatty acid-free BSA powder was dissolved in distilled water at a constant volume of 10 ml and 55°C water bath to prepare 10% BSA solution. Then, 1 ml of prepared 400 mM grazing acid solution was heated to 7 ml of 10% BSA solution, shaking and mixing to form PA with a final concentration of 50 mM. It was used in a sterile ultra-clean filter. After filtration and sterilization, they were separated into 1.5 ml sterile EP tube and stored at -80°C (Park et al., 2014).

### Cell Viability Assay and Safety Evaluation of CHF03

Methylthiazolyl diphenyltetrazolium bromide (MTT) was used to detect the cytotoxicity in response to CHF03 and PA. AML12 cells were plated into 96 well plates. After 24 h of starvation in serum-free medium, CHF03 and PA were added at different concentrations. After 24 h of incubation, 20 μl MTT soln was added to each well and incubated at 37°C for 4 hours. Culture medium was removed, and cells were then washed with DPB followed by addition of 100 ml DMSO to each well. Plates were shaken gently for 5 min. Absorption was measured at 450 nanometers with a miniature flat plate spectrophotometer (BD Biosciences, Franklin Lake, New Jersey, USA).

### Lipid Droplet Fluorescence Assay

The AML12 cells were treated with 0.6 mM PA for 24 hours. Lipid droplet fluorescence was evaluated by Bodipy 493/503 (Invitrogen Corporation) cell staining according to the manufacturer’s instructions. The images were obtained by confocal laser scanning microscopy (Leica TCS SP8; Leica, Wetzlar, Germany, 40 x 1.3 Nano-differential interference contrasts).

### Immunofluorescence Microscopy

Cells were immobilized in 4% paraformaldehyde for 20 min, then incubated with 10% l Triton X-100 for 10 min and washed with pre-cooled PBS solution 3 times. Cells were stained at room temperature with NF-κB p65 (1:1000; Cell Signal, cat#l8F5) and secondary antibodies binding to FITC A0568 (1:500, beyotime). The nuclei were stained with DAPI for 5 min and washed with PBS twice. The images were taken with an Olympus FV1000 confocal microscope.

### Western Blotting

The main antibodies used in the experiment were rabbit anti-mouse NF-κB p65 (1:1000; Cell Signaling, cat#L8F5), rabbit monoclonal antibody FASN (1:1000; cell Cell Signaling, Cat#C20G5), rabbit monoclonal antibody ACACA (1:1000; Abcam, Cat#EP687Y), and anti-mouse β-actin (3:5000; Sigma cat#A5441), goat anti-mouse or goat anti-mouse antibody labeled by HRP, rabbit or goat protection (3:5000; Santa Cruz). Lysates containing protease inhibitors were prepared by adding frozen RIPA buffer to liver or AML12 cell samples. Ultrasound cell crusher for 5 s was used to crush cells in each centrifugal tube. Samples were then centrifuged at 13,000 g for 15 min, and the clear supernatant collected and stored at -80°C. A BCA kit (Pierce BCA protein analysis kit; Thermo Science, Rockford, IL) was used to determine protein concentration. A protein sample of 20 μg was separated on the SDS polyacrylamide gel (10%) followed by transfer to a nitrocellulose membrane (thermosetting) and blocked for 1 h in phosphate-buffered saline containing Tween 20 (0.1%) and nonfat milk (5%). ImageJ software was used to analyze intensity and β-actin was used as the internal control.

### Quantitative Real-Time PCR

Total RNA was isolated from liver tissue or AML12 cells, and reverse transcription was carried out with 0.5 μg of total RNA using a retroviral transcription kit. The expression of RNA was quantified by using a LightCycler^®^ 480 detection system with SYBR Green I master (Mannheim Roche Applied Sciences, Germany) according to the manufacturer’s instructions. Target gene expression was analyzed with the 2^∆∆CT^ method, and β-actin was used as internal control. The primer sequences used to quantify gene expression are reported in **Table S2**.

### Flow Cytometry

The intracellular ROS was detected with the Reactive Oxygen Species Assay Kit (APPLYGEN, C1300). AML12 cell suspension was prepared by trypsin digestion, and then incubated with 2,7-dichlorofluorescein diacetate (DCFH-DA) at a final concentration of 10 mM for 20 min and washed with PBS. Following centrifugation, cell pellets were suspended in PBS for immediate analysis by flow cytometry. The data reported are the average of at least three independent experiments.

### Statistical Analysis

Data are expressed as means ± SD and analyzed by one-way ANOVA. Mean separation was by two-tailed Student’s *t*-test (Graph Pad Prism version 5 Software). Differences with *P* < 0.05 were considered statistically significant.

## Results

### Characteristics of Compounds From the CHF03

The LC-MS analysis was performed in negative and positive ion modes to obtain complete information about the chemical composition of CHF03. The peak MS spectrum is in **Figure S1**. All constituents had a full spectrum and were identified based on the database Metlin (https://metlin.scripps.edu). The identified compounds are reported in **Table S3** and **Table S4**.

### Treatment With CHF03 Reduced Body Weight Gain and Inhibited Steatosis of Liver

**Figure 1:**
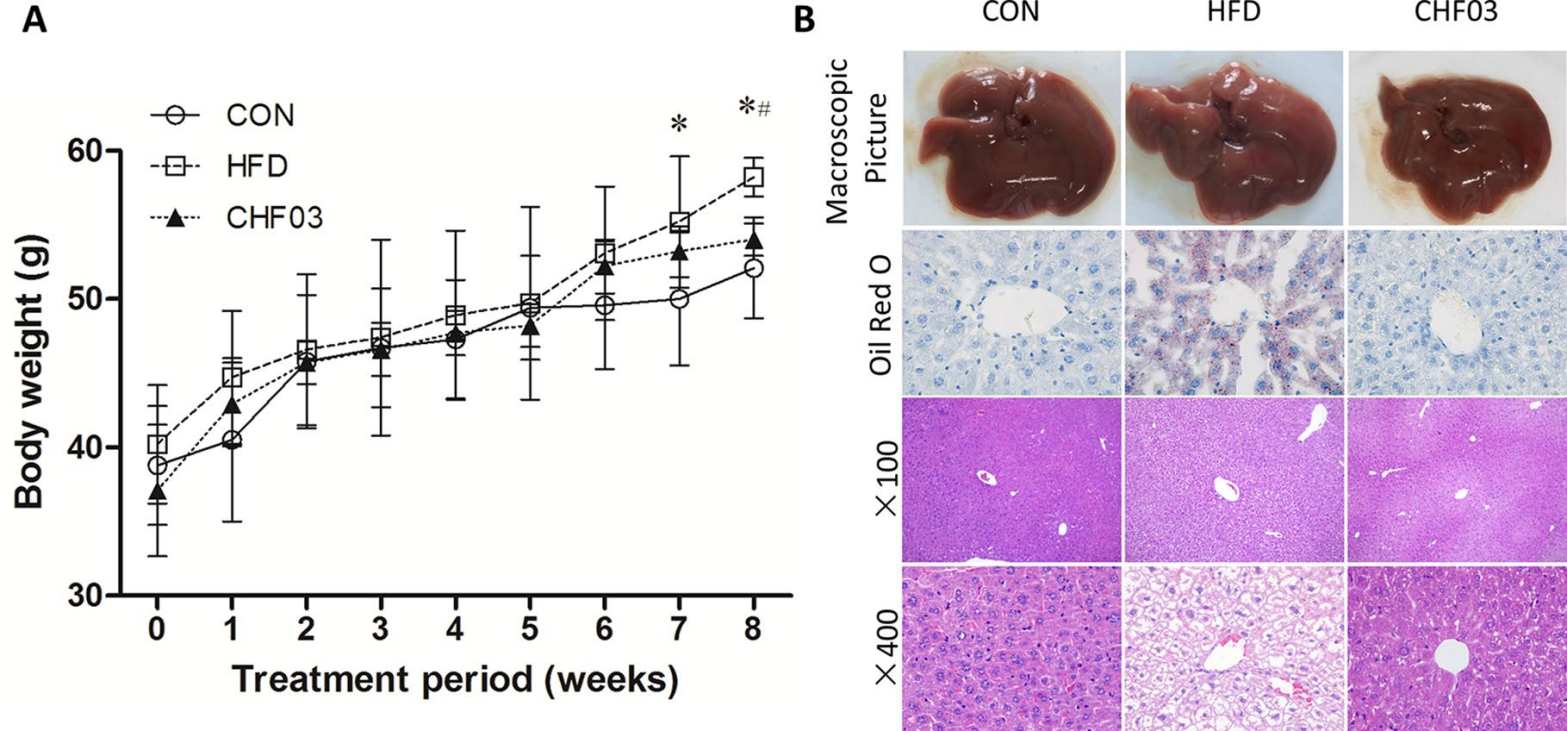
Changes in body weight and liver pathology. **(A)** Modulation of body weight by CHF03. A significant increase in body weight was observed at the 8th week after the successful establishment of the NAFLD model. After the 8 week CHF03 intervention, the CHF03 group exhibited a significant reduction in body weight compared with HFD group. **(B)** Representative macroscopic liver images, Oil Red O, and H&E-stained sections of liver tissues (original magnification ×100 and ×400). Lipid droplets are stained in orange. Macrovesicular and microvesicular steatosis is observed in the hepatic lobule of HFD group. Average body weights at the end of the treatment period were significantly different between the CHF03 and HFD groups (^#^ *p* < 0.05) and between the HFD and CON groups (*p < 0.05). Groups: CON, control; HFD, high-fat diet; CHF03, Chinese Herbal Formula.

After 8 weeks of treatment, the HFD mice had 117% greater body weight gain compared with controls (**Figure 1A**). In contrast, the CHF03 mice had 7% lower body weight compared with HFD mice. No difference of food intake was observed among experimental groups during the treatment (**Figure S2**). In the HFD group, liver tissue had a greater number of lipid droplets in hepatocytes (**Figure 1B**). Moreover, morphological observation indicated that the appearance of liver in mice fed HFD was hypertrophic and yellow (formed by fat accumulation), but the color, size, and weight of liver in mice receiving CHF03 was close to normal (**Figure 1B**). Under light microscopy, the liver tissue structure of the control group was normal, and the hepatic lobules were arranged neatly without droplets. In the NAFLD group, the hepatic lobules were arranged disorderly and the hepatocytes were steatotic. However, hepatic lobular injury and hepatocyte steatosis in CHF03 mice was not evident (**Figure 1B**). These results confirmed that CHF03 prevented the progression of weight gain and decreased the severity of HFD-induced NAFLD.

### Treatment With CHF03 Prevented Liver Injury and Increased Antioxidant Capacity

**Figure 2:**
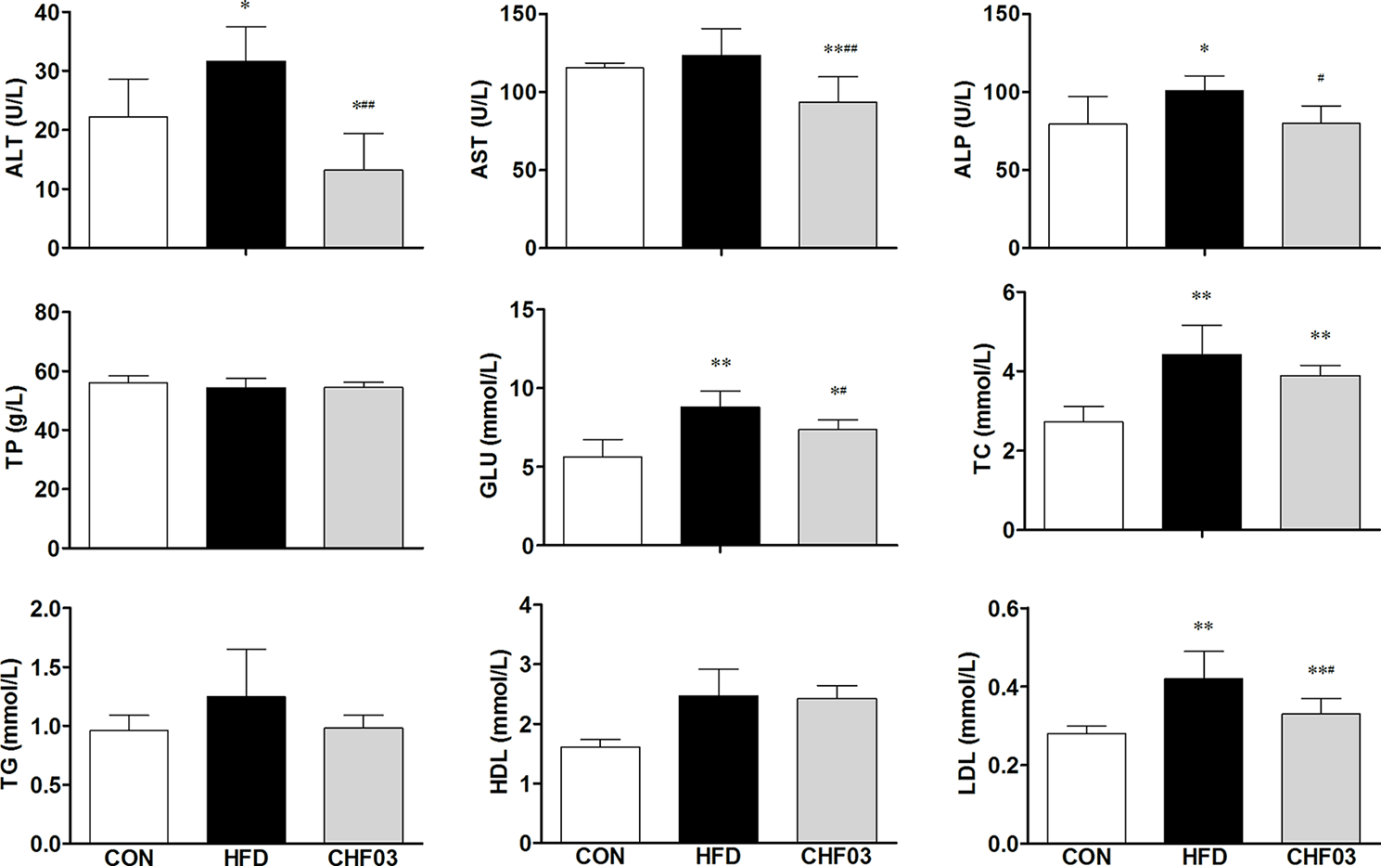
Serological profiles of ICR mice treated with CHF03. Alanine aminotransferase (ALT), Aspartate aminotransferase (AST), Alkaline phosphatase (ALP), Total protein (TP), Glucose (GLU), Total cholesterol (TC), Triglycerides (TG), High-density lipoprotein (HDL) and Low-density lipoprotein (LDL). All values are expressed as the mean ± SD (n = 6 per group). *p < 0.05, **p< 0.01 compared with CON; ^#^ *p* < 0.05, ^##^ *p* < 0.01 compared with HFD. Groups: CON, control; HFD, high-fat diet; CHF03, Chinese Herbal Formula.

**Figure 3:**
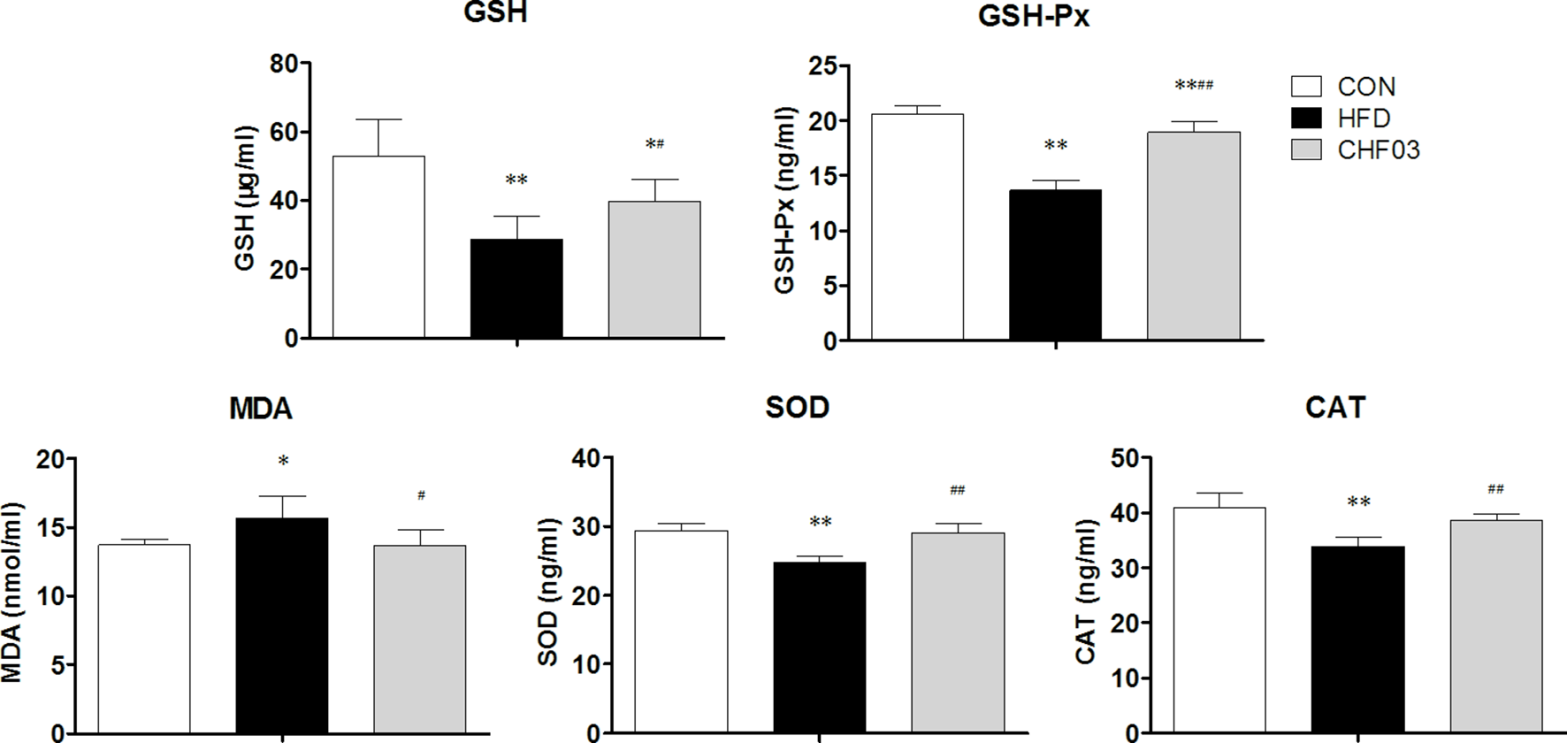
Oxidative stress evaluation in the liver of ICR mice treated with CHF03. Glutathione (GSH), Glutathione peroxidase (GSH-Px), Malondialdehyde (MDA), Superoxide dismutase (SOD) activity and Catalase (CAT) activity levels represents antioxidants enzymes and lipid peroxidation products in Liver. All values are expressed as the mean ± SD (n = 6 per group). *p < 0.05, **p < 0.01 compared with CON; ^#^ *p* < 0.05, ^##^ *p* < 0.01 compared with HFD. Groups: CON, control; HFD, high-fat diet; CHF03, Chinese Herbal Formula.

The effect of a high-fat diet on liver integrity is reported in **Figure 2**. The levels of ALT, ALP (*P* < 0.01), GLU, TC, and LDL were greater in the high-fat diet compared with the control group (*P* < 0.05). However, there was no significant difference in AST, TP, and HDL levels between the high fat diet group and the control group (*P* > 0.05). The levels of ALT, AST, ALP, GLU, TC, and LDL in CHF03 mice were lower significantly different from those in HFD mice (*P* < 0.05 or *P* < 0.01). The liver morphology of CHF03 mice was normal with lower liver damage compared with HFD group (**Figure 1B**). The levels of GSH, GSH-Px, MDA, SOD, and CAT are reported in **Figure 3**. The levels of GSH, GSH-px, SOD, and CAT in liver of mice fed the high-fat diet were lower compared with the control group (*P* < 0.01), while the concentrations of GSH, GSH-px, SOD, and CAT in CHFH03 mice were greater compared with mice fed the high-fat diet (*P* < 0.05 or *P* < 0.01). Compared with the control group, the MDA level of HFD mice in the high-fat diet group (*P* < 0.01) increased by 14%, while that of CHF03 group decreased by 12% (*P* < 0.01). The level of MDA in liver tissue decreased significantly when CHF03 was administered (*P* < 0.05). Untreated AML 12 cells showed PA-induced ROS production, while CHF03 treatment suppressed ROS production in a dose-dependent manner (**Figure S3**).

### Ultrastructural Liver Analyses

**Figure 4:**
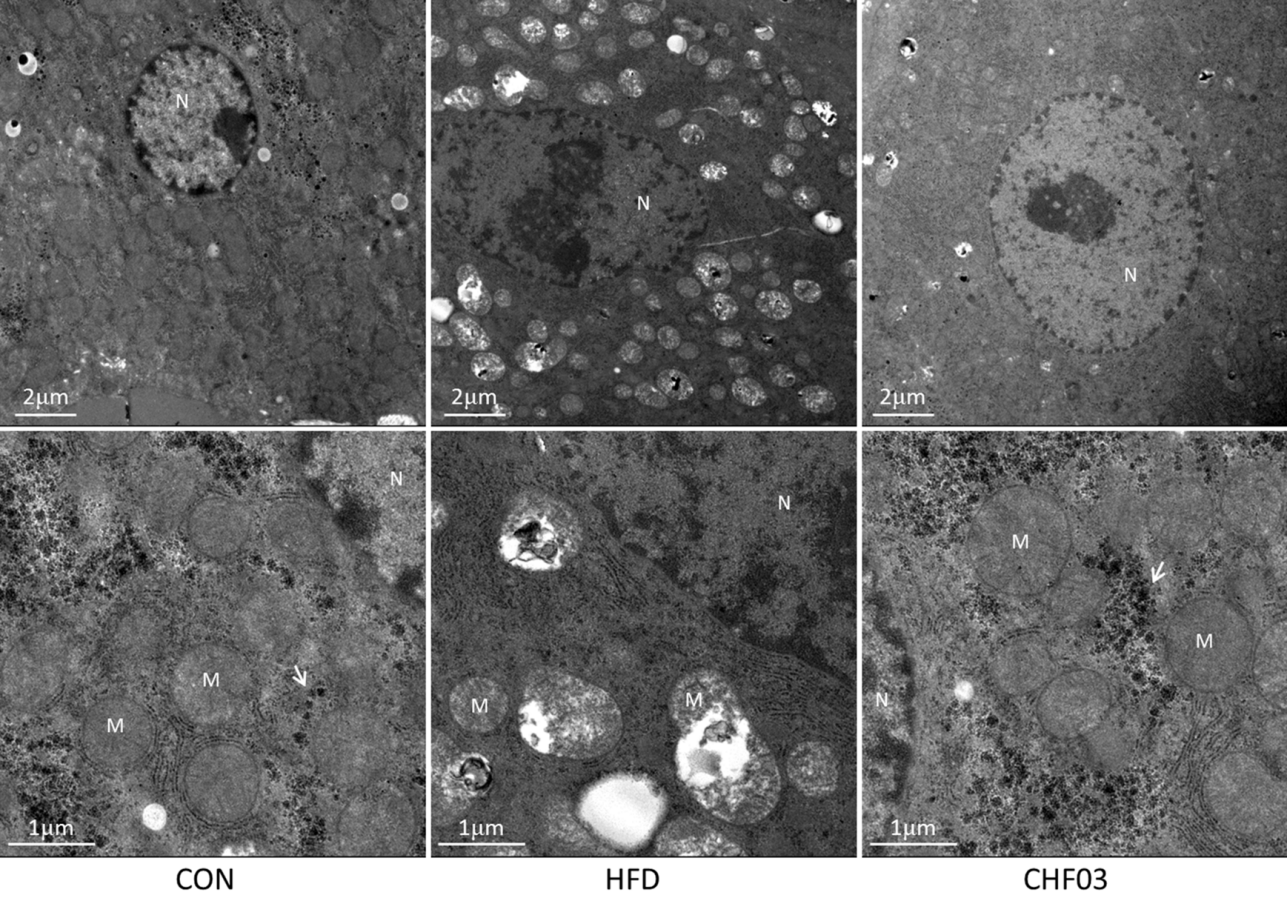
Ultra structural changes in the liver of ICR mice treated with high-fat. Panel with ultra-photomicrograph of hepatocytes showing the mitochondrial morphology of CON, HFD, and CHF03, respectively; M, mitochondria; N, nucleus. Whitehead arrows indicate glycogen. Magnifications: upper panel, 1700×; lower panel, 5000×. Groups: CON, control; HFD, high-fat diet; CHF03, Chinese Herbal Formula.

The mice fed with the high-fat diet for 8 weeks developed NAFLD, while administering CHF03 ameliorated hepatic steatosis and inflammation (**Figure 1B**). The electron microscopy analyses to assess hepatic mitochondrial morphology revealed well developed and organized mitochondria with an intact membrane and matrix in the control group. Liver cell structure appears normal, with round nuclei (**Figure 4**). In contrast, the HFD group had a decrease in mitochondrial matrix electron density and a loss of membrane integrity. Hepatocytes appeared swollen and there was evidence of cytoplasmic loosening, eosinophilic cytoplasm, reduced glycogen content, balloon-like degeneration, and steatosis. The membrane disruption led to mitochondrial matrix extravasation in the cytoplasm. Additionally, the HFD group had a remarkable disorganization of the cytoarchitecture (**Figure 4**). Ultrastructural analysis of mice with severe steatohepatitis due to feeding HFD revealed mitochondrial swelling with disruption of mitochondrial cristae. In contrast, despite mild steatohepatitis, mitochondria were less swollen with well-organized cristae in mice receiving CHF03 (**Figure 4**). Hepatocytes of animals treated with CHF03 exhibited numerous mitochondria and recovery of the mitochondrial membrane integrity and matrix electron density. Compared with the NAFLD group, hepatic cells from CHF03 mice maintained an organized ultra-structural arrangement with less glycogen accumulation. A decrease in lipid vesicle droplets was also observed in CHF03 compared with HFD mice (**Figure 1B**).

### CHF03 ATTENUATES mRNA and Protein Abundance of Lipid Metabolism and Inflammation Targets in Liver

**Figure 5:**
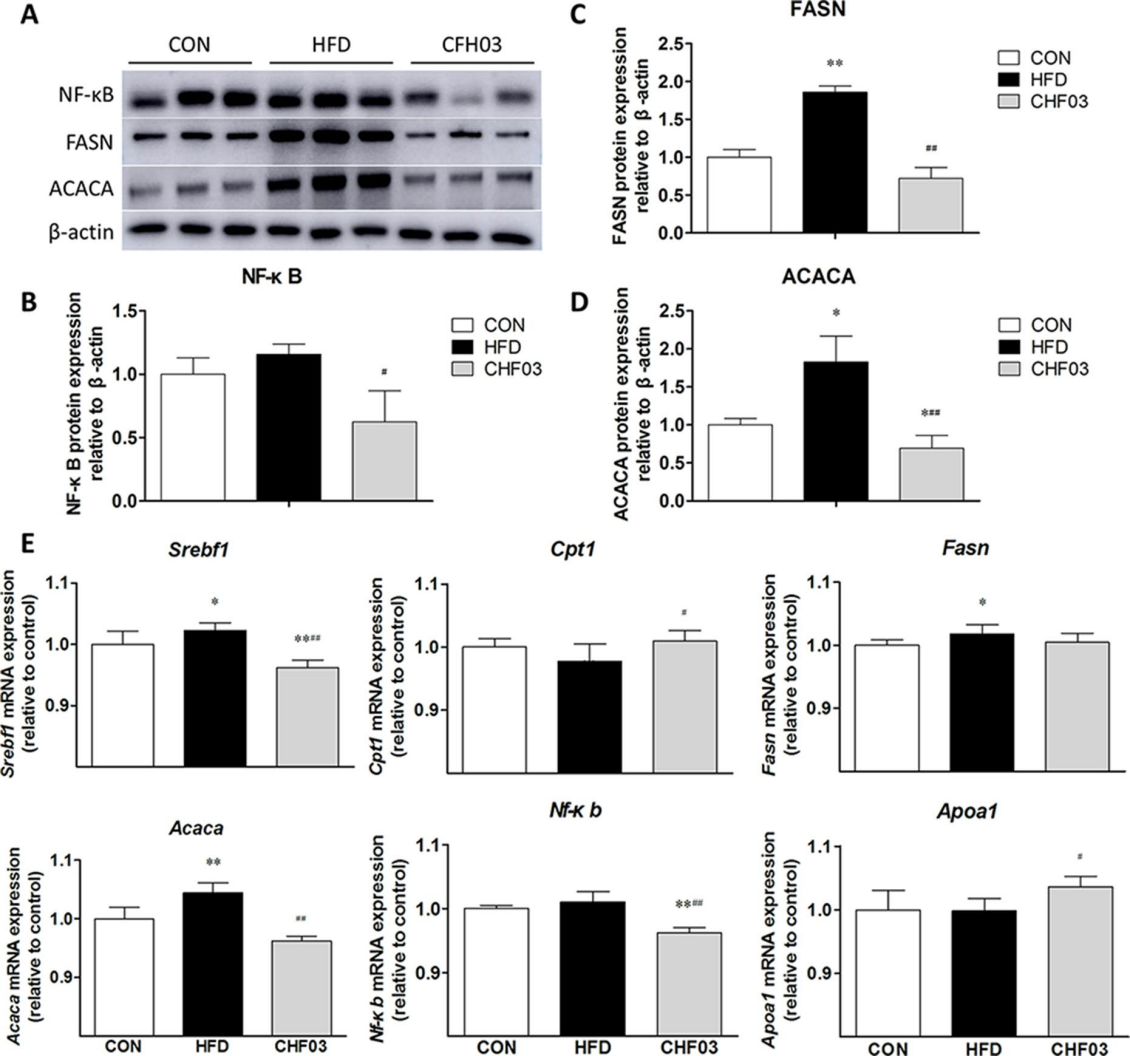
Effect of CHF03 on mRNA and protein abundance of key targets involved in lipid metabolism and inflammation in liver tissues of mice with NAFLD. **(A)** Abundance of nuclear factor κB (NF-κB), fatty acid synthase (FASN) and acetyl-CoA carboxylase (ACACA) in mouse liver detected by Western blot analysis. **(B**–**D)** Protein blot densities were quantified by ImageJ Software. Protein expression was analyzed by Western blot and normalized to β-actin. **(E)** mRNA abundance of lipid metabolism and inflammation genes in liver tissue. Sterol regulatory element binding transcription factor 1(SREBF1), carnitine palmitoyl transferase 1 (CPT1), fatty acid synthase (FASN), acetyl-CoA carboxylase (ACACA), nuclear factor κB (NF-κB) and apolipoprotein A1 (APOA1).Values are expressed as means ± SD. Values with different letters are significantly different in the groups (^#^ *p* < 0.05, ^##^ *p* < 0.01 compared with HFD; *p < 0.05, **p < 0.01 compared with CON). Groups: CON, control; HFD, high-fat diet; CHF03, Chinese Herbal Formula.

Compared with control mice, abundance of FASN (*P* < 0.01) (**Figure 5C**) and ACACA (*P* < 0.05) (**Figure 5D**) was greater in HFD mice, FASN (*P* < 0.01), and ACACA (*P* < 0.01) was lower in CHF03 compared with HDF mice (**Figure 5A**). The abundance of *Srebf1*, *Fasn*, and *Acaca* mRNA was induced in HFD mice. However, no induction of *Cpt1, Apoa1*, and *Nf-κb* was observed in HFD mice(**Figure 5B** and **Figure 5E**). In CHF03 mice all genes except *Cpt1* and *Apoa1* measured had lower (*P* < 0.05 or *P* < 0.01) abundance compared with HFD mice (**Figure 5E**).

### Effect of CHF03 on Cytotoxicity and Lipid Accumulation

**Figure 6:**
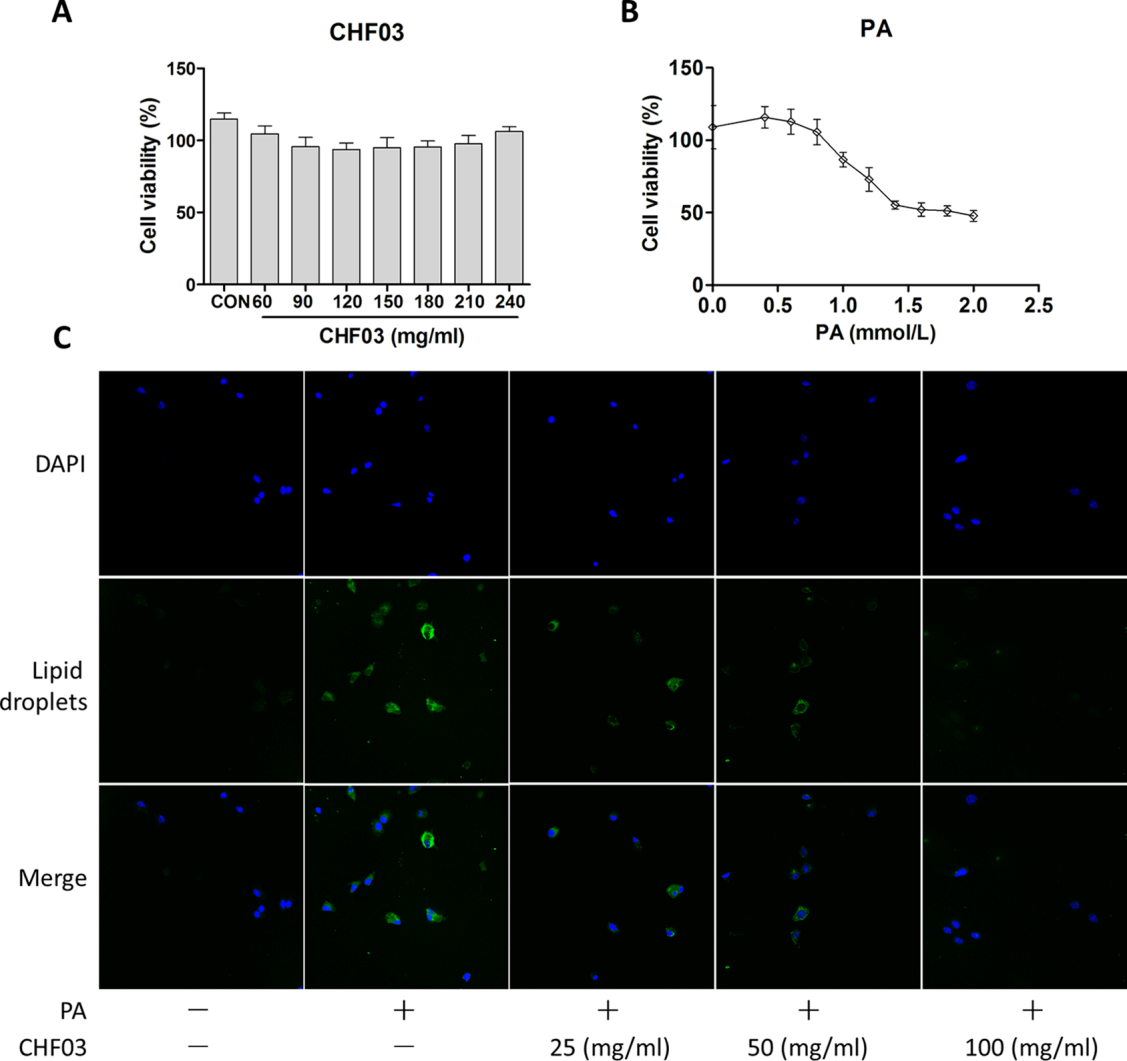
*In vitro* cytotoxicity and effect of CHF03 on lipid accumulation. **(A)** Effect of CHF03 on AML12 cell viability in response to different concentrations of CHF03 after incubation for 24 h. **(B)** Effect of PA on AML12 cell viability in response to different concentrations of PA after incubation for 24 h. Data represent mean ± SD of three independent experiments. **(C)** Effect of CHF03 on lipid accumulation in AML12 cells. Immunofluorescence images demonstrating nuclear staining (DAPI, Blue; Top images), lipid droplets staining (Green; middle images), and an overlay of both (merge, Bottom images) in AML12 cells incubated without or with PA and different concentration CHF03 (25, 50, and 100 mg/ml).

In order to exclude the inhibitory effect of CHF03 on lipid accumulation induced by cytotoxicity, cell viability tests were carried out 24 hours after treatment of AML12 cells with CHF03 of 240 mg/ml (**Figure 6A**). When cells were treated with 0.4, 0.6, 0.8, and 1 mM PA for 24 h, the inhibitory rate on cell viability was 115.8, 112.8, 105.6, and 86.7%, respectively, and its IC50 value was 1.8 mM (**Figure 6B**). Because a 24 h incubation with PA reduced cell vitality more than 50% at a concentration of 0.6 mM compared with control, we chose this concentration for subsequent experiments. As illustrated in **Figure 6C**, incubation with 0.6 mM PA for 24 h caused extensive lipid droplet formation.

### CHF03 Attenuates mRNA and Protein Abundance of Lipid Metabolism and Inflammation Targets in AML12 Cells

**Figure 7:**
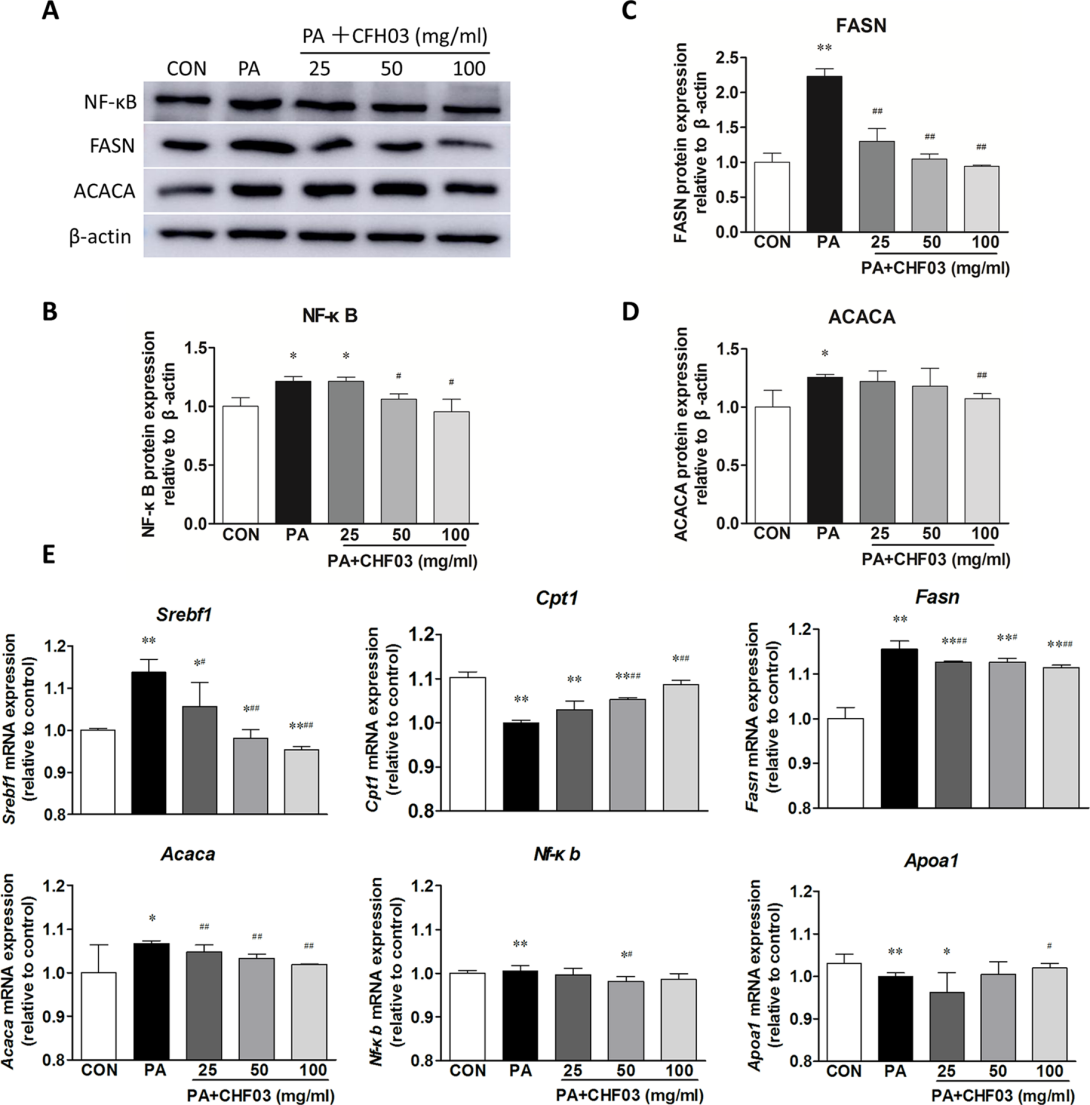
Effect of CHF03 on mRNA and protein abundance of targets involved in lipid metabolism and inflammation in AML12 cells. **(A)** AML12 cells were treated with various concentrations of CHF03 together with 0.6 mM/ml PA for 24 h. The abundance of NF-κB, FASN and ACACA in AML12 cells was analyzed by Western blot analysis. **(B**–**D)**. Protein blot densities were quantified by ImageJ Software. Protein expression was analyzed by Western blot and normalized to β-actin. **(E)** mRNA abundance of lipid metabolism and inflammation genes in AML12 cells. Sterol regulatory element binding transcription factor 1 (SREBF1), carnitine palmitoyl transferase 1 (CPT1), fatty acid synthase (FASN), acetyl-CoA carboxylase (ACACA), nuclear factor κB (NF-κB) and apolipoprotein A1 (APOA1).Values are expressed as means ± SD. Values with different letters are significantly different in the groups (^#^ *p* < 0.05, ^##^ *p* < 0.01 compared with HFD; *p < 0.05, **p < 0.01 compared with CON). Groups: CON, control; HFD, high-fat diet; PA, palmitic acid; CHF03 = Chinese Herbal Formula.

Compared with the control group, PA increased the protein abundance of NF-κB, FASN, and ACACA (**Figures 7A–D**), but supplementation with CHF03 attenuated this response reaching levels even lower than the control group (**Figure 7C**). Similarly, when compared with the control and PA treatment groups, supplementation with CHF03 also inhibited mRNA abundance of *Fasn* and *Acaca* in a dose-dependent manner (**Figure 7E**).

### Effect of CHF03 on Cellular Distribution of NF-κB

**Figure 8:**
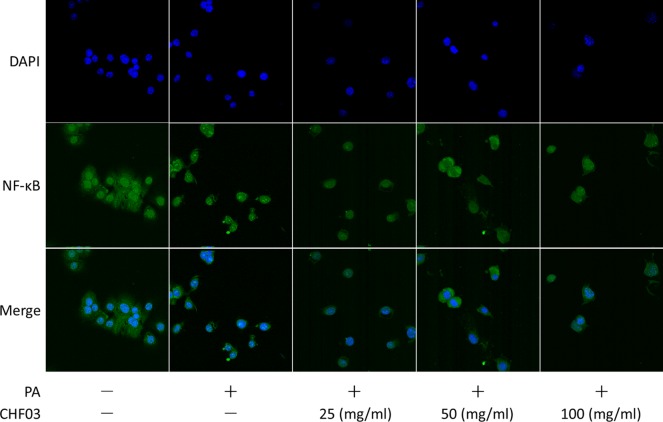
Effects of CHF03 on NF-κB activation in AML12 cells. NF-κB nuclear translocation was detected by confocal analysis using FITC A0568 -conjugated antibody for p65 subunit. AML12 cells (5 × 10^4^ cells) were treated with 0.6 mM PA in the absence or presence of CHF03 (25, 50, and 100 mg/ml) for 24 h.

Supplementation with CHF03 inhibited HFD-induced activation of NF-κB (**Figure 7**). Furthermore, CHF03 inhibited the translocation of NF-κB into the nucleus in PA-stimulated AML12 cells (**Figure 8**). Of these, CHF03 most potently inhibited the NF-κB translocation. On the whole, the effect of a CHF03-mix at a dose of 100 mg/ml was more effective than at 25 mg/ml, whereas the difference between the two groups was not significant.

## Discussion

A number of studies have revealed that traditional chinese medicine plays a unique role in alleviating chronic liver disease (Shergis et al., 2015), reducing lipidosis (Zhang et al., 2018), regulating immunity (Zhao et al., 2012), improving liver function, and protecting hepatocytes (Wat et al., 2018). Therefore, using 10 medicinal herbs we formulated CHF03 to test the hypothesis that it would exert protective effects against nafld *in vivo* in mice. Main components of CHF03 identified *via* LC/MS analysis included amino acids, organic Acids, fatty acids, nucleosides, and flavones. The two substances with the highest concentration were citric acid (40%) and L-Arginine (24%) (**Table S3** and **Table S4**). Our *in vivo* and *in vitro* studies revealed that oral CFH03 reduced HFD-induced lipid accumulation, chronic liver injury, and protected hepatocytes.

The increase of ROS can lead to lipid peroxidation, hence, oxidative stress is closely related to the pathogenesis of NAFLD (Mota et al., 2016). The enzymes SOD and GSH-px are important antioxidant enzymes that can prevent the damage of liver caused by reactive oxygen species (Gao et al., 2018) produced during oxidative stress. The MDA produced during lipid peroxidation can damage cell membranes and contribute to impaired cellular function (Perluigi et al., 2012). In addition, liver lipid accumulation accelerates the release of MDA and promotes nuclear mitochondrial DNA damage and apoptosis through the production of ROS. In this study, the increase in concentration of GSH-Px and SOD activity coupled with the decrease in MDA with CHF03 suggested an improvement of oxidative balance.

Excessive accumulation of neutral fat in hepatocytes due to the imbalance of lipid metabolism is a hallmark of NAFLD (Carr and Ahima, 2016). The onset of NAFLD can accelerate the onset of metabolic diseases such as obesity (Wang et al., 2010). In the process of cellular fatty acid uptake, fatty acids are first activated to form a fatty acyl CoA which can then enter the mitochondria for fatty acid beta-oxidation, with activity of CPT1 being rate-limiting in that process (Sun et al., 2018). From a pharmacological point of view, the use of drugs that can activate CPT1 increases the oxidation of fatty acids, thereby reducing the intracellular accumulation of these molecules (Dai et al., 2018). Apolipoprotein A1 (APOA1) is involved in regulating both lipid and energy metabolism, which may play important roles in liver regeneration, especially in cases of liver steatosis (Liu et al., 2019). An important cause for low circulating APOA1 levels is impaired liver function due in part to damage of hepatocytes (Trigueros-Motos et al., 2017).

Hepatic lipogenesis can be controlled by insulin signaling primarily through SREBF1 (Horton et al., 2002). The transcription factor promotes expression of several enzymes that are important for *de novo* lipogenesis, including ACACA and FASN (Browning and Horton, 2004). Recent data suggest that the activation of SREBF1 and, thus, lipogenesis is secondary to an ER stress in the steatotic liver (Ferre and Foufelle, 2010). Inhibition of ER stress in obese rodents decreases SREBF1 activation and lipogenesis and improves markedly hepatic steatosis (Kumar et al., 2019). Thus, the attenuation of hepatic lipid deposition along with upregulation of mRNA abundance of *Srebf1*, *Fasn*, *Acaca* and downregulation of *Cpt1* and *Apoa1* in response to supplementation with CFH03 underscored its potential for improving NAFLD.

The pathogenesis of NAFLD, inflammatory diseases and tumorigenesis (Robinson and Mann, 2010) includes activation of the NF-κB pathway (Lin et al., 2019). Mitochondrial dysfunction leads to oxidative imbalance causing lipid peroxidation and activation of NF-kB (Lu et al., 2015) with a consequent induction of pro-inflammatory cytokine synthesis and insulin resistance. Therefore, inhibiting the activity of NF-κB in NAFLD is one strategy to treat the disease. The fact that CHF03 supplementation decreased abundance of NF-κB *in vivo* and also in PA-stimulated AML12 cells suggests that the protective effect on NAFLD is related to its ability to regulate NF-κB signaling and inhibit inflammation.

## Conclusion

The protective effect of CHF03 on NAFLD in mice is related to its ability to regulate the inflammatory response and the lipogenic response controlled by SREBF1. Thus, CHF03 is a promising mix of natural compounds that could be used for the treatment of NAFLD.

## Data Availability Statement

The raw data supporting the conclusions of this manuscript will be made available by the authors, without undue reservation, to any qualified researcher.

## Author Contributions

CX and YC devised the study and will act as guarantors for the paper. YC, QW, CX, and TZ designed the research. XZ, RC, HG, and LH performed the research. YC wrote the first draft of the manuscript. JL revised the final manuscript as submitted. All authors read and approved the final manuscript.

## Conflict of Interest

The authors declare that the research was conducted in the absence of any commercial or financial relationships that could be construed as a potential conflict of interest.

## Abbreviations

CHF, Chinese herbal formula; NAFLD, nonalcoholic fatty liver disease; NF-κB, nuclear factor-kappa B; LC/MS, liquid chromatography-mass spectrometry; GSH, Glutathione; GSH-px, glutathione peroxidase; MDA, malondialdehyde; SOD, Superoxide dismutase; CAT, catalase; HFD, high-fat diet; ACACA, acetyl-CoA carboxylase; FASN, fatty acid synthase; SREBF1, sterol regulatory element binding transcription factor 1; CPT1, carnitine palmitoyl transferase 1; APOA1, apolipoprotein A1; ROS, reactive oxygen species; TBARS, traditional Chinese medicines; thiobarbituric acid reactive substances; TEM, transmission electron microscope.
