# Supplementary material for: Chinese Herbal Formula (CHF03) Attenuates Non-Alcoholic Fatty Liver Disease (NAFLD) Through Inhibiting Lipogenesis and Anti-Oxidation Mechanisms
Source: Front Pharmacol. 2019 Oct 15;10:1190. doi: 10.3389/fphar.2019.01190 (PMC6803500; doi:10.3389/fphar.2019.01190)
Supplement: Supplementary file 1 [file DataSheet_1.docx]

**Supplementary Figure**

**
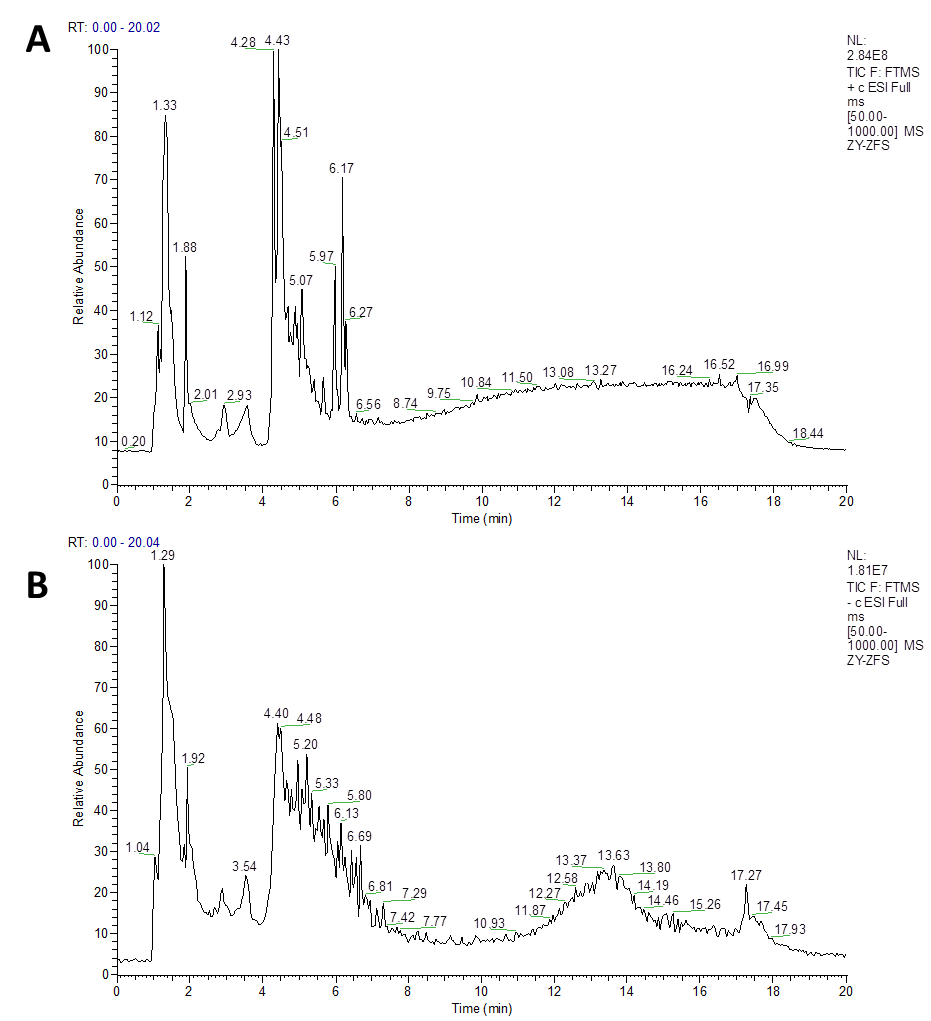
**

**Supplementary Figure S1 The Total Ion Chromatogram of CHF03.** (A) The Total Ion Chromatogram of CHF03 (ESI +). (ESI+) represents the positive ion detection mode, in which the mass analyzer scans only positive charged ions and filters out negative charged ions to obtain positive charged ions information during the detection process; (B) The Total Ion Chromatogram of ZFS (ESI-). (ESI-) denotes the negative ion detection mode, in which the mass analyzer scans only negative charged ions and filters out positive charged ions, thus obtaining the information of negative charged ions.


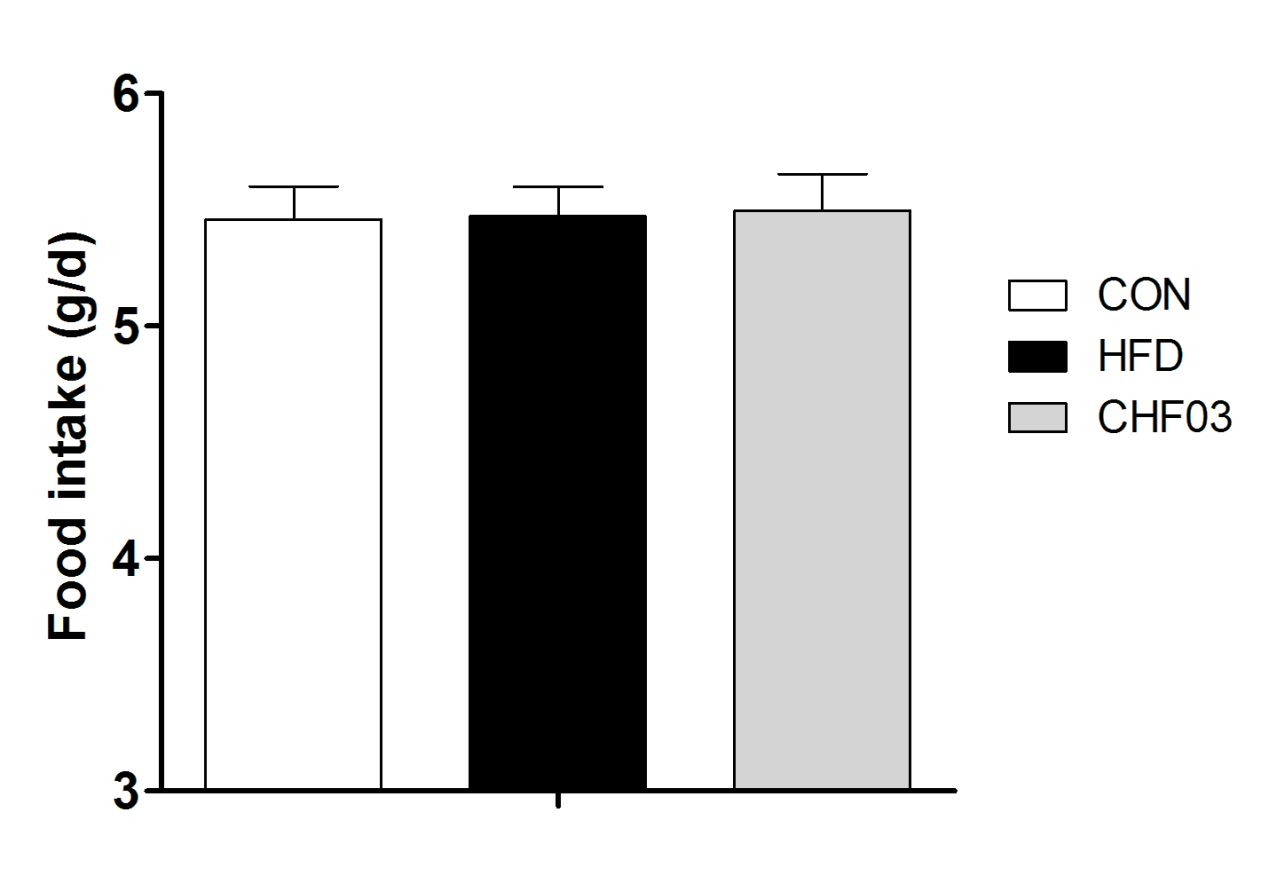


**Supplementary Figure S2 Changes in food intake.** All values are expressed as the mean ± SD (n=6 per group). * *p* < 0.05, ** *p*< 0.01 compared with CON; ^#^ *p* < 0.05, ^##^ *p* < 0.01 compared with HFD. Groups: CON = control; HFD = high-fat diet; CHF03 = Chinese Herbal Formula.

**
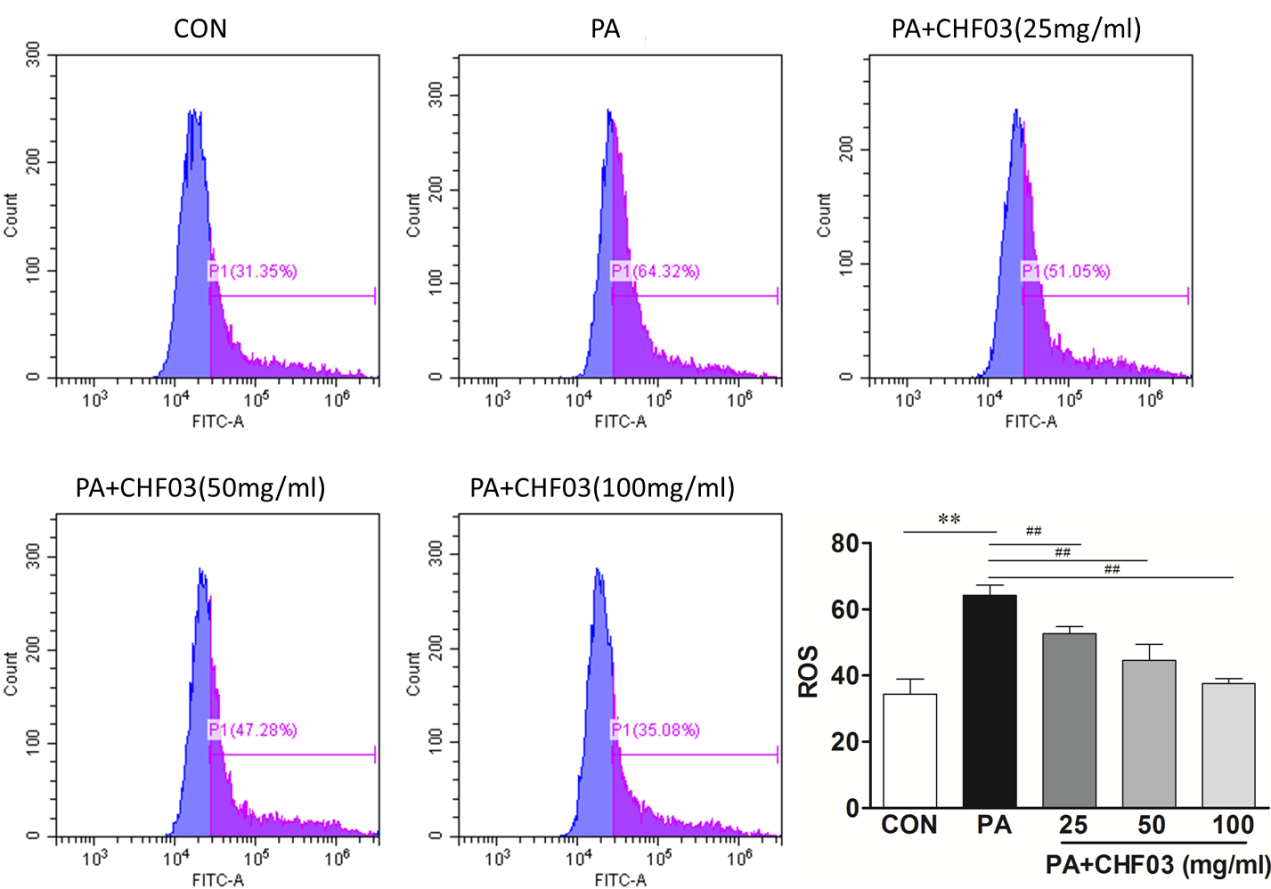
**

**Supplementary Figure S3 CHF03 inhibits PA-induced reactive oxygen species (ROS) production in AML 12 cells cells.** Values are expressed as means ± SD. Values with different letters are signiﬁcantly different in the groups (^#^ *p* < 0.05, ^##^ *p* < 0.01 compared with HFD; ^*^ *p* < 0.05, ^**^ *p* < 0.01 compared with CON). Groups: CON = control; PA = palmitic acid; CHF03 = Chinese Herbal Formula.

**Supplementary Table**

**Supplementary Table S1 Medicinal herbs included in Chinese Herbal Formula (CHF03)**

| Scientific Name | Chinese Name | Latin Name | Amount (g) |
| --- | --- | --- | --- |
| Pilose Asiabell Root | Dang Shen | *Radix Codonopsitis Pilosulae* | 30 |
| Largehead Atractylodes Rh | Bai Zhu | *Rhizoma Atractylodis Macrocephalae* | 12 |
| Plantain Seed | Che Qian Zi | *Semen Plantaginis* | 15 |
| Cablin Potchouli Herb | Huo Xiang | *Herba Agastachis Seu Pogostemi* | 10 |
| Indian Buead | Fu Ling | *Glabrous greenbrier rhizome* | 15 |
| South Dodder Seed | Tu Si Zi | *Cuscuta chinensis Lam* | 12 |
| Rehmannia Root | Shu Di | *Rehmannia glutinosa (Gaertn.) DC* | 15 |
| Tangerine Peel | Chen Pi | *Pericarpium Citri Reticulatae* | 10 |
| Barbed Shullcap Herb | Ban Zhi Lian | *Scutellaria barbata D. Don* | 15 |
| Liquoric Root | Gan Cao | *Glycyrrhiza uralensis Fisch. ex DC* | 10 |
| Total | | | 144 |

**Table S2 Primer sequences used in mRNA quantitation by reverse transciption-polymerase chain reaction.**

| Gene | Forward primers | Reverse primers |
| --- | --- | --- |
| *Srebf1* | 5′-GATGTGCGAACTGGACACAG-3′ | 5′-CATAGGGGGCGTCAAACAG-3′ |
| *Cpt1* | 5′-ATGACCTCCTGGCATTCTCC-3′ | 5′-AGGACCCTGAGGCATCTATT-3′ |
| *Fasn* | 5′-GCTGGCATTCGTGATGGAGTCGT-3′ | 5′-AGGCCACCAGTGATGTAACTC-3′ |
| *Acaca* | 5′-TAATGGGCTGCTTCTGTGACTC-3′ | 5′-CTCAATATCGCCATCAGTCTTG-3′ |
| *Nf-ĸb* | 5′-GCTCCTGTTCGAGTCTCCATG-3′ | 5′-CATCTGTGTCTGGCAAGTACTGG-3′ |
| *Apoa1* | 5′-GGCACGTATGGCAGCAAGAT-3′ | 5′-CCAAGGAGGAGGATTCAAACTG-3′ |
| *β-actin* | 5′-GAGACCTTCAACACCCCAGC-3′ | 5′-ATGTCACGCACGATTTCCC-3′ |

**Supplementary Table S3 Chemical components identiﬁed from CHF03 by high-performance liquid**

**chromatography-electrospray ionization/mass spectrometry (ESI +)**

| Name | Rt [min] | Molecular Weight | CAS | Content (ng/μL) |
| --- | --- | --- | --- | --- |
| L-Phenylalanine | 2.983 | 165.0785 | 63-91-2 | 130.78 |
| L-Arginine | 1.201 | 174.111 | 74-79-3 | 423.03 |
| L-Tyrosine | 1.397 | 181.0732 | 60-18-4 | 3.46 |
| L-Glutamate | 1.313 | 147.0525 | 56-86-0 | 0.79 |
| L-Isoleucine | 2.072 | 131.0941 | 61-90-5 | 85.70 |
| L-Lysine | 1.16 | 146.1049 | 56-87-1 | 2.56 |
| L-Proline | 1.341 | 115.0628 | 147-85-3 | 143.07 |
| Pyroglutamic acid | 1.98 | 129.042 | 98-79-3 | 22.54 |
| ferulic acid | 4.69 | 194.0573 | 1135-24-6 | 17.13 |
| Sinapic acid | 4.66 | 224.0675 | 530-59-6 | 0.90 |
| Styrene | 6.038 | 104.0621 | 100-42-5 | 0.34 |
| Chorismic acid | 3.906 | 226.0474 | 617-12-9 | 0.06 |
| m-Coumaric acid | 4.64 | 164.0467 | 588-30-7 | 6.99 |
| 1,2,3-Trihydroxybenzene | 3.232 | 126.0311 | 533-73-3 | 7.44 |
| Caffeic Acid | 4.48 | 180.0415 | 4607-41-4 | 0.98 |
| Thymol | 4.451 | 150.1038 | 89-83-8 | 0.23 |
| Adenosine | 1.965 | 267.0958 | 58-61-7 | 42.61 |
| Adenine | 1.957 | 135.0546 | 73-24-5 | 4.22 |
| Guanosine | 1.96 | 283.0908 | 118-00-3 | 2.98 |
| Guanine | 1.441 | 151.0487 | 73-40-5 | 9.96 |
| cAMP | 1.473 | 329.0502 | 60-92-4 | 15.08 |
| Quercetin 3-galactoside | 4.491 | 464.0946 | 482-36-0 | 8.03 |
| Arcapillin | 5.282 | 360.0832 | NA | 0.54 |
| Glyceollin | 5.928 | 338.1144 | NA | 3.83 |
| Isorhamnetin | 4.954 | 316.0571 | 480-19-3 | 2.66 |
| Malvidin | 5.267 | 330.0727 | 643-84-5 | 1.62 |
| Naringenin | 5.177 | 272.0675 | 480-41-1 | 5.36 |
| Quercetin | 4.988 | 302.0415 | 117-39-5 | 6.72 |
| Quercetin 3-(3-p-coumaroylglucoside) | 4.653 | 610.1301 | 76211-70-6 | 0.36 |
| Rhamnetin | 5.528 | 316.0572 | 480-19-3 | 2.02 |
| Taxifolin | 4.405 | 304.0572 | 480-18-2 | 0.47 |
| Cyanidin 3-O-rutinoside | 4.334 | 594.1558 | 28338-59-2 | 9.61 |
| Diosmetin | 5.179 | 300.0621 | 520-34-3 | 45.26 |
| Eriodictyol | 4.51 | 288.0621 | 552-58-9 | 7.01 |
| Genistein | 4.478 | 270.0516 | 446-72-0 | 77.55 |
| Genistin | 4.476 | 432.1037 | 529-59-9 | 9.13 |
| Luteolin | 4.806 | 286.0465 | 491-70-3 | 81.34 |
| Pelargonidin 3-O-(6-O-malonyl-β-D-glucoside) | 4.525 | 518.1035 | 165070-68-8 | 0.79 |
| Pelargonidin 3-O-rutinoside | 4.389 | 578.1612 | NA | 4.66 |
| Petunidin 3-O-glucoside | 4.537 | 478.1092 | 6988-81-4 | 3.66 |
| Quercitrin | 4.503 | 448.0987 | 522-12-3 | 13.78 |
| Sakuranin | 4.532 | 448.1351 | NA | 17.95 |
| Scutellarein 5-glucuronide | 4.501 | 462.0778 | NA | 114.11 |
| Naringin | 4.482 | 580.1763 | 10236-47-2 | 6.90 |
| Gallocatechin | 1.388 | 306.0707 | NA | 0.12 |
| Peonidin 3-rhamnoside 5-glucoside | 13.76 | 609.1748 | 53859-11-3 | 0.31 |
| Hesperetin | 4.538 | 302.0778 | 520-33-2 | 67.93 |
| 2-Hexyl-3-phenyl-2-propenal | 5.773 | 216.1506 | 101-86-0 | 9.00 |
| DL-pipecolic acid | 1.925 | 129.0785 | 535-75-1 | 5.81 |
| Hydroquinidine | 4.963 | 326.1984 | 1435-55-8 | 1.62 |
| Hypoxanthine | 1.963 | 136.0379 | 68-94-0 | 1.03 |
| Trigonelline | 1.584 | 137.0471 | 535-83-1 | 19.23 |
| Xanthosine | 4.474 | 284.0787 | 146-80-5 | 13.73 |
| Caffeine | 4.413 | 194.0837 | 58-08-2 | 2.68 |
| D-Mannitol | 1.231 | 182.0785 | 69-65-8 | 0.22 |
| a-L-Rhamnose | 1.239 | 164.0679 | 6014-42-2 | 0.07 |
| Gibberellin A53 | 5.402 | 348.1923 | NA | 0.72 |
| Glutinosone | 5.699 | 220.1455 | 55051-94-0 | 3.89 |
| Plaunol B | 4.789 | 356.1247 | 69749-00-4 | 2.36 |
| Quillaic acid | 6.58 | 486.3329 | 631-01-6 | 1.20 |
| Genipin | 4.406 | 226.083 | 6902-77-8 | 0.18 |
| Medicagenic acid | 6.215 | 502.327 | 599-07-5 | 0.15 |
| p-Cymene | 4.894 | 134.1089 | NA | 1.12 |
| Pantothenic Acid | 3.524 | 219.1103 | 137-08-6 | 11.34 |
| Pyridoxine | 2.326 | 169.0736 | 65-23-6 | 1.09 |
| Pyridoxal | 3.258 | 167.0579 | 66-72-8 | 1.86 |
| Niacin | 5.633 | 123.0314 | 59-67-6 | 0.49 |
| Niacinamide | 1.985 | 122.0473 | 98-92-0 | 13.27 |
| Palmitic amide | 9.57 | 255.2558 | 629-54-9 | 11.40 |
| 13Z-Docosenamide | 13.06 | 337.3334 | 112-84-5 | 17.68 |
| Oleamide | 9.873 | 281.2709 | 301-02-0 | 29.22 |
| Stearamide | 12.982 | 283.2865 | 124-26-5 | 1.22 |
| Coumarin | 5.111 | 146.0362 | 91-64-5 | 0.54 |
| 3 Hydroxycoumarin | 3.902 | 162.0309 | 939-19-5 | 4.77 |
| Scopoletin | 4.766 | 192.0414 | NA | 4.49 |
| Benzoic acid | 4.7 | 122.0362 | 65-85-0 | 1.74 |
| α-ketoisovaleric acid | 1.86 | 116.0469 | 759-05-7 | 2.71 |
| Succinic acid | 1.957 | 118.0273 | 110-15-6 | 16.60 |
| nandrolone | 5.468 | 274.1923 | 434-22-0 | 3.05 |
| α-Linolenic Acid | 7.357 | 278.224 | 463-40-1 | 4.11 |
| Butyric acid | 1.866 | 88.0521 | 107-92-6 | 4.94 |
| LysoPC(16:0) | 7.257 | 495.3313 | NA | 3.66 |
| MG(0:0/18:3/0:0) | 6.214 | 352.2602 | NA | 0.78 |
| Indoleacrylic acid | 4.278 | 187.0625 | 1204-06-4 | 58.10 |
| Methyl cinnamate | 3.805 | 162.0675 | 103-26-4 | 0.68 |
| 5-Hydroxy-L-tryptophan | 2.276 | 220.0845 | 4350-09-8 | 1.48 |
| Indoleacetaldehyde | 2.371 | 159.0681 | NA | 8.06 |
| Acetylcholine | 2.005 | 145.1099 | 51-84-3 | 0.31 |
| Cinnamic acid | 3.612 | 148.0521 | 621-82-9 | 11.45 |
| Gingerol | 5.765 | 294.182 | 58253-27-3 | 1.16 |
| Hippuric acid | 4.356 | 179.0576 | 495-69-2 | 0.43 |
| Jasmolone | 5.898 | 180.1144 | 54383-66-3 | 2.27 |
| (-)-Jasmonic acid | 5.713 | 210.1247 | 6894-38-8 | 4.45 |
| Indole | 4.301 | 117.0573 | 120-72-9 | 25.72 |
| Methyl jasmonate | 4.519 | 224.1403 | 39924-52-2 | 4.83 |
| Phenylacetic acid | 4.746 | 136.0518 | 103-82-2 | 4.73 |
| acetophenone | 4.403 | 120.0568 | 98-86-2 | 1.12 |
| Choline | 9.289 | 103.0991 | 62-49-7 | 0.09 |
| Tropic acid | 4.458 | 166.065 | 552-63-6 | 11.08 |

**Supplementary Table S4 Chemical components identiﬁed from CHF03 by high-performance liquid**

**chromatography-electrospray ionization/mass spectrometry (ESI -)**

| Name | Rt [min] | Molecular Weight | CAS | Content (ng/μL) |
| --- | --- | --- | --- | --- |
| L-Isoleucine | 2.06 | 131.09469 | 61-90-5 | 131.79 |
| L-Phenylalanine | 2.933 | 165.07893 | 63-91-2 | 1325.78 |
| Pyroglutamic acid | 1.991 | 129.04272 | 98-79-3 | 880.82 |
| L-Cystine | 4.179 | 240.02653 | 56-89-3 | 10.62 |
| Chlorogenic Acid | 4.127 | 354.09478 | 327-97-9 | 37.47 |
| ferulic acid | 4.705 | 194.0574 | 1135-24-6 | 126.95 |
| Sinapic acid | 4.68 | 224.06787 | 530-59-6 | 42.31 |
| 1,2,3-Trihydroxybenzene | 3.154 | 126.03172 | 533-73-3 | 156.26 |
| Caffeic Acid | 3.013 | 180.04208 | 4607-41-4 | 10.15 |
| Gallic acid | 3.708 | 170.02138 | 149-91-7 | 16.84 |
| Gentisic acid | 3.623 | 154.0266 | 490-79-9 | 230.92 |
| Shikimic acid | 1.836 | 174.05273 | 138-59-0 | 9.61 |
| Homogentisic acid | 3.694 | 168.04204 | 451-13-8 | 101.13 |
| m-Coumaric acid | 4.65 | 164.04712 | 588-30-7 | 231.05 |
| Syringic acid | 2.887 | 198.05249 | 530-57-4 | 6.29 |
| Salicylic acid | 4.496 | 138.03141 | 69-72-7 | 84.81 |
| Uridine | 2.02 | 244.06907 | 58-96-8 | 56.52 |
| Inosine | 1.276 | 268.07889 | 58-63-9 | 23.15 |
| IMP | 4.452 | 348.04661 | 131-99-7 | 13.75 |
| cAMP | 1.971 | 329.05183 | 60-92-4 | 14.50 |
| Diosmetin | 5.179 | 300.06245 | 520-34-3 | 283.61 |
| Genistein | 4.566 | 270.05208 | 446-72-0 | 39.99 |
| Malvidin | 5.272 | 330.07307 | 643-84-5 | 15.51 |
| Naringenin | 5.185 | 272.06776 | 480-41-1 | 159.60 |
| Quercetin | 5.038 | 302.04179 | 117-39-5 | 102.40 |
| Cyanidin 3-O-rutinoside | 4.326 | 594.15626 | 28338-59-2 | 128.75 |
| Isorhamnetin | 4.948 | 316.05741 | 480-19-3 | 30.50 |
| Luteolin | 4.861 | 286.04682 | 491-70-3 | 1233.00 |
| Pelargonidin 3-O-rutinoside | 4.944 | 578.16133 | NA | 2.81 |
| Petunidin 3-O-glucoside | 4.585 | 478.10942 | 6988-81-4 | 98.94 |
| Quercitrin | 4.555 | 448.09913 | 522-12-3 | 264.69 |
| Dihydromyricetin | 4.479 | 320.05192 | 27200-12-0 | 6.05 |
| Eriodictyol | 4.523 | 288.06209 | 552-58-9 | 109.70 |
| Naringin | 4.499 | 580.17667 | 10236-47-2 | 323.44 |
| Quercetin 3-(3-p-coumaroylglucoside) | 4.67 | 610.12941 | 76211-70-6 | 10.64 |
| Quercetin 3-galactoside | 4.519 | 464.09335 | 482-36-0 | 725.72 |
| Scutellarein 5-glucuronide | 4.502 | 462.07786 | NA | 555.04 |
| Taxifolin | 4.43 | 304.05702 | 480-18-2 | 10.72 |
| Rutin | 4.428 | 610.14931 | 153-18-4 | 40.79 |
| Hesperetin | 4.523 | 302.07789 | 520-33-2 | 288.21 |
| Purine | 1.299 | 120.04223 | 120-73-0 | 972.07 |
| 2-Furoic acid | 1.439 | 112.01615 | 88-14-2 | 1082.60 |
| Caffeine | 4.492 | 194.08423 | 58-08-2 | 15.66 |
| D-Glucarate | 1.543 | 210.03737 | 87-73-0 | 55.67 |
| D-Glucuronic acid | 1.264 | 194.04247 | 6556-12-3 | 514.29 |
| Glutaric acid | 1.311 | 132.04226 | 110-94-1 | 2402.13 |
| L-Xylulose | 1.458 | 150.05294 | 527-50-4 | 138.62 |
| D-Mannitol | 1.265 | 182.07878 | 69-65-8 | 121.53 |
| Gluconic acid | 1.299 | 196.058 | 526-95-4 | 1865.63 |
| α-D-Glucose | 1.307 | 180.06317 | 492-62-6 | 4477.77 |
| α,α-Trehalose | 1.738 | 342.1154 | 57-50-1 | 881.56 |
| Raffinose | 4.067 | 504.16731 | 512-69-6 | 34.02 |
| Genipin | 4.414 | 226.08368 | 6902-77-8 | 6.78 |
| Gibberellin A12 | 8.093 | 332.19787 | NA | 90.26 |
| Medicagenic acid | 6.193 | 502.32825 | 599-07-5 | 48.18 |
| Quillaic acid | 6.564 | 486.33328 | 631-01-6 | 56.94 |
| Rishitin | 7.443 | 222.16141 | 18178-54-6 | 50.38 |
| Gibberellin A17 | 4.924 | 378.1664 | 18411-79-5 | 68.34 |
| Gibberellin A36 | 5.465 | 362.17181 | NA | 148.81 |
| Ganoderic acid H | 17.348 | 572.2945 | 98665-19-1 | 33.48 |
| Geranyl diphosphate | 4.391 | 314.06284 | 763-10-0 | 2834.61 |
| Pantothenic Acid | 3.485 | 219.1103 | 137-08-6 | 439.36 |
| Riboflavin | 4.246 | 376.1359 | 83-88-5 | 33.93 |
| Sulfuric acid | 1.575 | 97.96744 | 7664-93-9 | 2415.23 |
| Phosphoric acid | 1.471 | 97.97696 | 7664-38-2 | 409.67 |
| Benzoic acid | 4.717 | 122.03673 | 65-85-0 | 293.35 |
| Citric acid | 1.446 | 192.02674 | 77-92-9 | 11316.82 |
| Lactic acid | 2.959 | 90.0318 | 50-21-5 | 265.29 |
| Pyruvate | 1.45 | 88.01615 | 127-17-3 | 634.89 |
| Hexadecanedioic acid | 5.656 | 286.21382 | NA | 23.18 |
| Quinic acid | 4.373 | 192.06302 | 77-95-2 | 72.02 |
| Aconitic acid | 2 | 174.0164 | 499-12-7 | 466.57 |
| Itaconic acid | 2.512 | 130.02669 | 97-65-4 | 143.73 |
| Maleic acid | 1.996 | 116.01102 | 110-16-7 | 324.93 |
| Malic acid | 1.879 | 134.02155 | 6915-15-7 | 2324.74 |
| Oxoglutaric acid | 1.487 | 146.02162 | 328-50-7 | 30.04 |
| Succinic acid | 2.072 | 118.02664 | 110-15-6 | 1074.09 |
| Glyceric acid | 1.354 | 106.02678 | 473-81-4 | 43.52 |
| nandrolone | 5.46 | 274.19264 | 434-22-0 | 4.67 |
| α-Linolenic Acid | 7.321 | 278.22397 | 463-40-1 | 226.80 |
| LysoPC(15:0) | 7.22 | 481.31539 | NA | 100.96 |
| Traumatic Acid | 5.273 | 228.13561 | 6402-36-4 | 59.10 |
| acetophenone | 4.646 | 120.05742 | 98-86-2 | 180.62 |
| Citramalic acid | 1.499 | 148.03727 | 2306-22-1 | 653.04 |
| Mevalonic acid | 3.028 | 148.07363 | 150-97-0 | 116.72 |
| Phenylacetic acid | 4.741 | 136.05243 | 103-82-2 | 97.44 |
| (-)-Jasmonic acid | 5.711 | 210.12533 | 6894-38-8 | 220.53 |
| Malonic acid | 1.474 | 104.0111 | 141-82-2 | 843.94 |
| Xanthoxin | 6.275 | 250.15644 | 8066-07-07 | 94.46 |
| Gentisin | 4.621 | 258.05214 | 437-50-3 | 22.59 |
| Tropic acid | 4.432 | 166.06257 | 552-63-6 | 1632.97 |
| Xanthoxic acid | 9.992 | 266.15443 | NA | 4.18 |
